# Social control of egg-laying in independently nest-founding bumble bee queens

**DOI:** 10.1186/s12862-025-02364-0

**Published:** 2025-04-09

**Authors:** Blanca R. Peto, Claudinéia P. Costa, Meghan E. Moore, S. Hollis Woodard

**Affiliations:** Department of Entomology, University of California, Riverside, Riverside, CA USA

**Keywords:** Bumble bee, Queens, Reproduction, Social regulation

## Abstract

**Background:**

Evolution has shaped diverse reproductive investment strategies, with some organisms integrating environmental cues into their reproductive decisions. In animal societies, social cues can further influence reproductive decisions in ways that might support the survival and success of the social group. Bumble bees are a lineage of eusocial insects wherein queens initiate nests independently. Bumble bee queens enter their eusocial phase only after successfully rearing their first offspring and thereafter exhibit an increased rate of egg-laying. We tested the idea that during bumble bee nest initiation, queen reproduction is socially context-dependent and under the control of social conditions in the nest.

**Results:**

Our findings reveal that in the bumble bee *Bombus impatiens*, queen egg-laying follows a dynamic, stereotypical pattern and is also heavily influenced by social group members. During the initial stages of nest initiation, accelerated egg-laying in queens is associated with the presence of workers or older larvae and pupae. Moreover, workers are required for queens to maintain increased levels of egg laying across the nest initiation stage. We also confirmed a previously-described pattern where queens temporarily decelerate egg-laying early in nest-founding, only to increase it again when the first adult workers are soon to emerge. This “pause” in egg-laying was observed in all *B. impatiens* queens as well as in additional species examined.

**Conclusions:**

Our results support the idea that eusocial systems can employ socially context-dependent control of queen egg-laying as a reproductive strategy. In some solitary-founding lineages, including bumble bees, queens may reach their full reproductive potential only after the emergence of the first adult workers, who then take over brood care. This stands in contrast to the hyper-reproductivity observed in some eusocial species. The presence of workers and older brood (who will soon eclose) not only alleviates queen brood care responsibilities but may also provide signals that cause queens to increase their reproductive output. These phenomena may allow queens to adapt their reproductive output to the conditions of the colony. Broadly, these findings highlight the dynamic interplay between social conditions and reproduction in bumble bees.

**Supplementary Information:**

The online version contains supplementary material available at 10.1186/s12862-025-02364-0.

## Background

Evolution has shaped reproductive investment strategies in diverse ways. Organisms can integrate information from their surrounding environment (such as food availability and environmental stability) into decisions about whether to invest in the production of offspring or other non-reproductive processes [1–6]. Environmentally-responsive reproduction is thought to be adaptive. Investing too heavily in offspring production at times when resources are too limited or might be better-directed to other processes, can lead to fitness costs [3]. These fitness costs can come in the form of not producing the maximum number of viable offspring.

For animals that live in social groups, the social environment adds additional dimensions to how offspring investment is mediated. In some eusocial insect systems, where helpers are present at incipient nest stages and are present continuously (honey bees, swarm-founding wasps, non-solitary founding ants), queens can spend much of their lives laying eggs at a high rate. In contrast, the apex of reproductive output is reached more gradually in eusocial insect systems where queens establish nests independently. These queens are the sole caretakers in incipient nests and must provide continuous care for developing offspring until they reach adulthood. Queens that found nests independently [7–9] carry out all care-related tasks alone, including feeding and (in some systems) incubation, which are essential for the early growth and development of their young [10, 11]. Once workers emerge, however, the queen is relieved of providing brood care, as the workers take over the bulk of responsibilities. Consequently, a queen’s ability to effectively allocate resources before a worker emerges might significantly impact her personal fitness. The success of a queen and her survival in this critical early nest founding stage directly impacts whether the colony progresses to produce workers and eventually reproductive offspring [12–14].

One of the hallmarks of nesting success is the first emergence of offspring, which reflects a successful transition from the subsocial to eusocial stage. From here on, social group members in the nest have myriad opportunities to influence and mediate one another’s investment strategies. Social regulation is exemplified in eusocial insects, which live in groups with overlapping generations—where parents coexist with adult offspring—and engage in collective alloparental care. In these groups, reproduction is suppressed in most female offspring (workers) and typically monopolized by one or a few females (queens) within the nest [15–17]. Queens inhibit reproduction in workers through mechanisms such as pheromones and dominance interactions [18–21].

Queen control of worker reproduction has been studied extensively [18, 19, 22, 23]. There is also some limited evidence for the converse: that queen reproduction can be under the control of social group members. Support for this phenomenon has been found thus far in multiple ant lineages, Vespine and *Polistes* wasps, and in bumble bees. Within these systems, the presence and number of workers [7, 24–27], and even the presence of developing brood [28], can increase queen oviposition rates at the onset of nest initiation. Offspring presence is hypothesized to allow queens to accelerate their egg-laying at a time that coincides with an increasing number of helpers (workers) in the nest, who will assume and specialize on brood care tasks and allow the queen to allocate resources to reproduction [26]. It is currently unclear if this reallocation is permanent, or if queens will revert to earlier, lower egg-laying rates without sustained social conditions. Exploring the under-researched phenomenon of worker control of, and support for, queen reproduction will help illuminate a crucial aspect of eusociality: that in some systems, social group members facilitate reproduction, rather than suppressing it.

Here, we used the bumble bee *Bombus impatiens* (genus *Bombus*, family Apidae) to test the idea that in this independently-founding eusocial insect lineage, queen reproduction is under the control of multiple signals that are indicative of helpers or the potential for future helpers in the nest. Specifically, we tested whether both adult workers who provide brood care and late-stage brood, which will develop into care-providing workers within days, positively influence queen egg-laying. There is some prior evidence of the positive effects of both workers [25, 26] and older brood [28, 29–31] on queen reproduction, but they have not been examined together. The impact of these social group members has also not been examined across the full course of the nest initiation period. Previous natural history observations have noted dynamic egg-laying patterns in early nests of bumble bee queens that can only be partially explained by social regulation. Early observations of bumble bee nests from Alford [32] state that “*once the initial egg clump is completed*,* further eggs are not normally laid until the first brood reaches the pupal stage*.” A similar phenomenon was reported by Sladen [12] and Heinrich [10]. Thus, we also sought evidence for this phenomenon that to our knowledge has not been empirically examined. We collected quantitative information about whether queens do temporarily decelerate and then reaccelerate their reproductive rate very early in nest-founding, with the latter in association with social conditions in the nest.

We confirmed that the pattern of temporarily reduced egg laying, previously described by Alford [32] and others [10, 12], occurs in *B. impatiens*, and provide some preliminary evidence that it might occur in other bumble bee species. We believe that this biological phenomenon is a consistent behavioral pattern exhibited by bumble bee queens such that the queen can more effectively allocate resources between current brood and investment in her own future reproductive potential. Without this pattern, the queen would potentially face fitness costs by providing time and energy-intensive care to an over-abundance of offspring, which may be beyond her capacity to care for. By temporarily reducing laying eggs, queens can instead allocate more consistent care to existing offspring in the nest, which may increase overall nest fitness. We found that patterns of queen egg laying are heavily influenced by social group members. Queens increased their reproductive output with both the presence of group members that assist with brood care (adult workers) or the artificial addition of group members who will soon do so (older larvae and pupae). Furthermore, we found that the presence of workers is necessary to maintain higher queen reproductive output. These findings overall support the broad idea that eusocial systems can employ socially context-dependent control of queen egg-laying as a reproductive strategy during early nest establishment and growth.

## Methods

### Experiment 1a: egg laying across early nest development

***B. impatiens *rearing**: Fourteen mature *B. impatiens* colonies (consisting of a queen and workers, with queen pupae present) were provided by Koppert Biological Systems (Howell, MI, USA) and maintained within their commercial boxes at the University of California, Riverside’s Entomology Building. Colonies were stored at 23 °C and 60–70% relative humidity (r.h.) under constant darkness or occasional red light, which bees cannot see. Individual adult callow queens < 24 h old (*n* = 62), identified by their silvery appearance and inability to fly, were removed from natal colonies. These queens were then placed in individual cups (Hi-Plas plastic container, 7.5 cm in diameter) and fed *ad libitum* with mixed-source honey bee-collected pollen (Koppert Biological Systems) and artificial nectar following the recipe in Boyle et al. [33]. At age three days, queens were placed daily in mating cages (BugDorm-6E620 Insect Rearing Cage, 60 × 60 × 120 cm) between 7 and 9 h per day under constant ambient light and allowed to mate for an additional seven days (until age ten days), until they were observed copulating and were presumed to have successfully mated [34–36]. Adult males (≥ 24 h old) were procured from five natal colonies and were mated with queens from different natal colonies. While in the mating cage, all queens and males received pollen and artificial nectar, as previously described. Queens were singly mated and were not subjected further to mating cages after mating occurred. All nests produced worker offspring, indicating that all queens used in this experiment were successfully mated and were able to produce diploid (female) offspring. A subset of nests (*n* = 36) also produced male offspring (average of 2.97 males per nest). Male offspring were removed from all future offspring analyses, due to low volumes.

Following mating, queens were placed in new individual containers with pollen and artificial nectar and kept in an Invictus Drosophila Incubator maintained at 27 °C and 60% relative humidity. At adult ages 12 and 13 days, all queens were temporarily removed from the incubator and treated with CO_2_ gas for 30 min. This treatment causes queens to bypass diapause [37] but does not otherwise change the course of nest initiation [37–40]. After the second day of CO_2_ treatment, queens were placed into plastic nest cages (Biobest Group USA, Inc.; dimensions: 17 cm x 17 cm x 9.5 cm) provided with pollen, a honey-bee wax-covered pollen ball (to provide readily accessible wax), and artificial nectar. These cages were transferred to the University of California, Riverside’s Insectary and Quarantine Facility and kept under 24-hour darkness except for brief exposure to light when food was changed and when photographs were taken. At this stage and for the remainder of the experiment, the nests were maintained at 28 °C and 60% r.h. Across the experiment, pollen was replenished every 5–7 days and the artificial nectar solution was replaced every two weeks to prevent spoilage. A total of 12 queens were removed from this experiment because they either died before initiating a nest (*n* = 8) or the first day of egg-laying was not observed (*n* = 4).

#### Nest monitoring for eggs

Nests were visually inspected once daily for the presence of eggs and any new eggs observed in nests were assigned to the day they were observed. When queens lay eggs at the earliest stage in nest development, they produce an open waxen cup that eggs are deposited into, then they close it. These egg cups appear as a small, raised mound of wax measuring approximately 6 × 5 mm and standing about 4 mm high [32], typically located either on the pollen provision [10, 12, 41], on a central plastic mound within each nest (in the boxes used for this experiment), or on existing brood (as colonies develop), as seen in Supplementary Fig. 2.

#### Nest imaging and daily egg-laying rates

Beginning on the day the first egg cup was observed in a nest box, we photographed nests nearly every day for the subsequent 44 days. This timeframe encompasses the emergence of the first cohorts of workers and the period when queens likely transition from providing most brood care in the nest to predominantly egg-laying [25, 26, 39, 42]. Photographs were taken between 1000 and 1200 h and individual nests were exposed to light for a limited time (10 min maximum per day) during this process. From the photographs, we counted the total number of egg cups present in the nest and noted their location. This allowed us to calculate the daily increase in egg cups within each nest, accounting for the egg cup rearrangement that bumble bees sometimes do. Occasionally, nests were not photographed on a given day, but these gaps were never longer than three days when this happened (< 10 instances), except for only one nest where no photos were taken during the last four days of the experiment (42–45), but the number of egg cups was still noted visually. When days were missed, we averaged the total new egg cups equally across days to provide an approximate number of new egg cups per day. In some of our early nests, we noted instances of egg cup destruction either through egg pulling or egg eating by the queen. If queens appeared to restart their nests (noted by the disappearances of the first few cups and subsequent appearance of egg cups in a new location) nest initiation was dated as the first new day of egg-laying.

**Table 1:** Summary of experiments examining queen egg-laying dynamics and nest development. N refers to the number of queens or nests involved in the experiments. See Fig. 1 for more details

Phenomenon	Experiment	Question	*N*=	Response
Dynamic patterns of queen egg-laying across early nesting period	1a	5-day intervals and patterns of egg-laying	62	number of egg cups
Dynamic patterns of queen egg-laying across early nesting period	3	Proportion of ovaries resorbed in relation to treatment	62	proportion of ovaries resorbed
Influence of brood on queen egg-laying	4	Brood addition and timing of re-initiation	30	number of days until re-initiation
Influence of brood on queen egg-laying	1a	Differences between brood type in nests (pause entry)	62	oldest brood type present in nest
Influence of brood on queen egg-laying	1a	Differences between brood type in nests (pause exit)	62	oldest brood type present in nest
Association between the pause and nest developmental characteristics	1a	Length of the pause and number of days until the first worker emergence	62	number of days until the first brood emergence
Association between the pause and nest developmental characteristics	1a	Length of the pause and number of workers within one week	62	number of workers within one week
Association between the pause and nest developmental characteristics	1a	Length of the pause and the body size of all workers after emergence	62	body size of workers after emerging
Queen egg-laying is also under the control of adult workers in the nest	2a, 2b	Differences in the number of egg clutches after worker emergence	30	total number of egg cups
Queen egg-laying is also under the control of adult workers in the nest	2a	Number of brood in nests and presence/ absence of workers in the nest	30	total number of brood

**Fig. 1:**
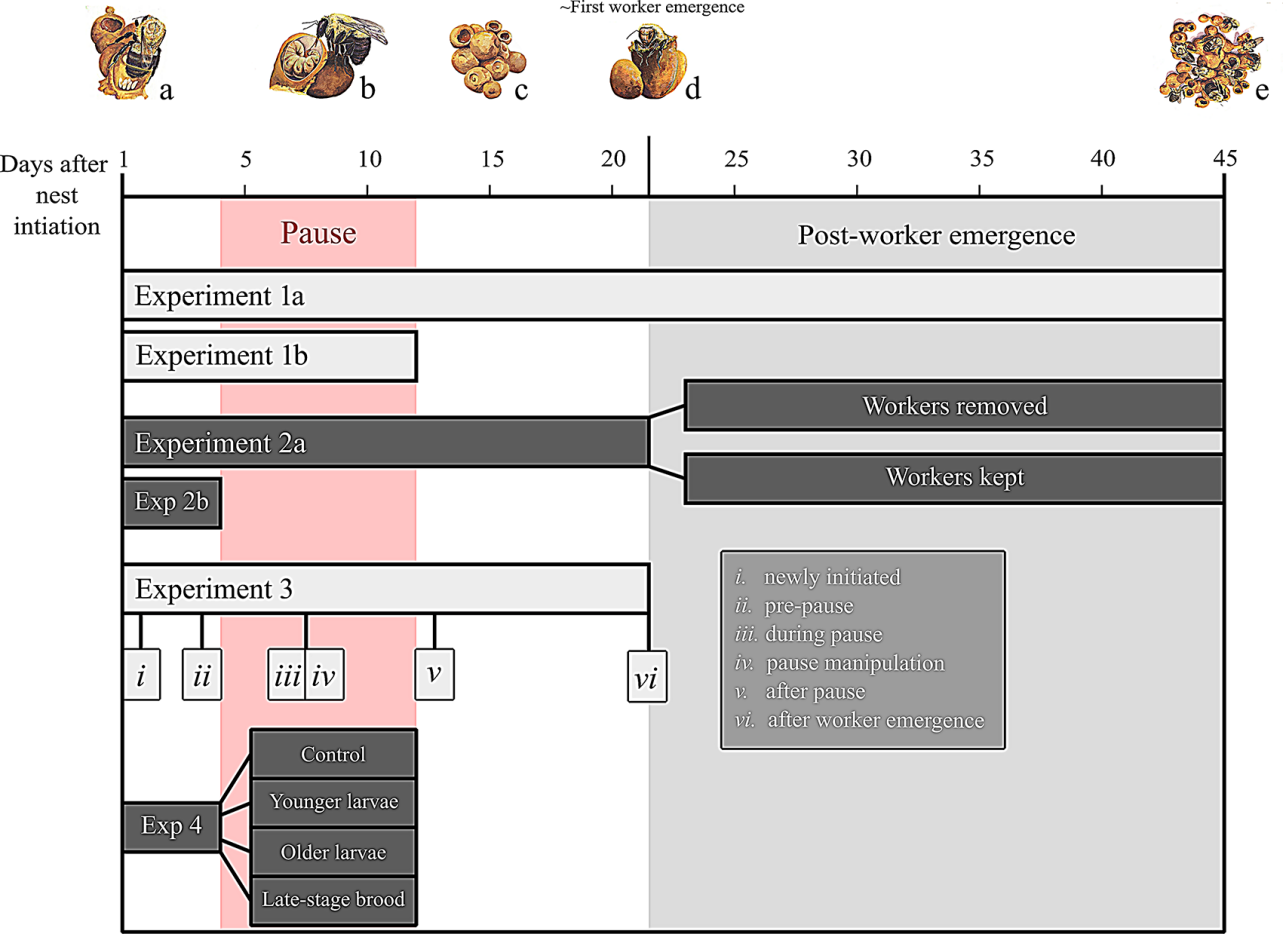
Experimental timeline. Parts a-e of the figure refer to different colony developmental stages: **a**) nest initiation, **b**) pause **c**) reinitiation **d**) first worker emergence **e**) late-colony development

### Experiment 1b: patterns of egg-laying in additional bumble bee species

We used a similar methodology to Experiment 1a (but simplified) to perform a small observational study examining egg laying in a set of queens of additional, opportunistically collected species: *Bombus vosnesenskii*, *n* = 6, *Bombus mckayi*, *n* = 2, *Bombus frigidus*, *n* = 1 (see Fig. 1; Table 1). This was done to validate (yes/no) whether evidence for the pause (see definition below) can be found in species outside of *B. impatiens.* In this observational study, we only tracked queen egg laying until queens resumed laying after the pause. *B. vosnesenskii* queens were hand-netted in the wild from the San Bernardino National Forest in California. After collection, queens were kept cool on ice during transport and maintained at the University of California, Riverside’s Insectary and Quarantine Facility using methods similar to those previously described for *B. impatiens*. *B. mckayi* and *B. frigidus* queens were collected from Anchorage, Alaska and reared using similar methods at the University of Alaska Anchorage. Queens were monitored daily until the presence of the first egg cup in the nest was recorded, then egg laying was subsequently monitored only until queens paused, then resumed egg laying.

### Experiment 2a: impact of adult workers on queen egg laying

Following the same methods as above (for Experiment 1a) for mating, housing, nest imaging/monitoring, and quantifying egg laying, we examined if the presence of workers is necessary for sustained reproductive acceleration in queens, and whether it supports nest development (see Fig. 1; Table 1). A total of 30 queens from four natal colonies were used for this experiment and separated into two treatment groups: eclosed workers kept in their nest (worker+, *n* = 10) and eclosed workers removed from their nests (worker-, *n* = 20). For the worker- group, all newly-eclosed adult offspring were removed on the day of eclosion and stored at -80 C. In this experiment, we assume that any eggs after worker emergence in the nest were laid by the queen. This is because, at the earliest stages of nest development, which we examined here, queens strongly inhibit worker egg-laying [43, 44]. Moreover, we did not see an increase in the number of egg cups in our worker + nests following the emergence of workers beyond that of the observed capacity of queens in the beginning of the experiment, which would be predicted if workers were laying eggs in the nests.

### Experiment 2b: eggs per cup before pause

We used an additional separate, smaller set of *B. impatiens* queens (*n* = 10) to determine how many eggs are present within egg cups (which requires destructive sampling) at the onset of nest initiation. Queens were collected from five natal colonies and mated with males from four natal colonies. Otherwise, we used similar rearing methods as described above but collected queens four days after the first eggs were observed in the nest (see Fig. 1; Table 1). Queens were frozen for at least 48 h before dissections. We then dissected the nests and counted the number of eggs within each egg cup. These data were used to understand how the number of eggs per cup differs between the earliest and latest stages of nest development (based on a comparison to data from queens in Experiment 2a). Additionally, we used these data to explore whether the number of eggs per cup is influenced by whether workers are left in nests (worker+) or removed on the day of their emergence (worker-).

### Nest dissections

For experiments examining nest development and brood (Experiments 2, 3, and 4), the nests were frozen and subsequently dissected on dry ice. The total number of egg cups and brood were counted. We used visual inspections of brood to divide each individual into four different categories corresponding to one or more stages characterized by Rozen et al. [41]: (i) eggs; (ii) younger larvae (corresponding to 1st and 2nd instars, housed in communal chambers and fed as a group); (iii) older larvae (corresponding to 3rd and the majority of the 4th instar, larvae begin spinning a cocoon); and (iv) and late-stage brood (which includes the post-defecation stage of the fourth instar, prepupae, and pupae). We followed this same characterization for the brood addition experiment (see below, Experiments 3 and 4) to determine the oldest brood type at re-initiation.

### Body size measurements

We measured the body size of all queens and adult worker offspring in all experiments using the Minitool Measuring Scale (in mm). The length of the second marginal cell of both wings was measured and averaged for each bee as a proxy for body size. In bumble bees, the second marginal cell is highly correlated with overall body size [45].

### Experiment 3: ovarian development and resorption across nest development

To examine how patterns of ovarian resorption and development change across early nest development, a set of queens (*n* = 73) were collected from five natal colonies and mated with males from six natal colonies, and reared using the same methods as outlined above. Of these original 73 queens, 11 died or failed to initiate within an appropriate timeframe (within a ~ 2-month period) and were removed from the experiment. The remaining 62 queens were then distributed as equally as possible (with respect to their natal colony) among the following six treatment groups. The six treatments included: (i) *newly initiated* (collected on the second day of egg-laying after initiation), (ii) *pre-pause* (collected the day before queens entered the pause period, i.e., the fourth day after nest initiation; timing determined based on data from our primary experiment to determine an appropriate time for this treatment); (iii) *during pause* (collected three days into the pause, also based on data from the primary experiment); (iv) *pause manipulation* (a combination of older larvae and pupae brood were added in clumps of ~ 3–7 brood items four days into the “pause” period) (see Fig. 1; Table 1). The *pause manipulation* treatment group was designed to test whether queens develop their ovaries prematurely during the pause if brood are introduced; more information about this methodology and its rationale is provided below; (v) *after pause* (collected five days after queens re-initiating egg-laying) and (vi) *after worker emergence* (collected 10 days after the first worker emerged). Queens were collected on dry ice to preserve tissue integrity for ovary dissections.

Two aspects of ovarian development were considered for all queens (*n* = 62): levels of resorption and lengths of the terminal oocytes. Ovary resorption status (resorbed or unresorbed) was qualitatively scored for each of the eight terminal oocytes. Resorbed oocytes are identifiable by their yellow coloring and misshapen appearance [46] and were removed from oocyte length analyses because resorption can result in misshapen oocytes with unreliable length measurements. We then measured the lengths of the eight terminal, unresorbed oocytes using methods such as Duchateau & Velthuis [46] and Medler [45].

### Experiment 4: impact of artificial brood addition on queen egg-laying

A set of 48 queens (from two natal colonies, mated with males from seven colonies) was used to test whether the addition of brood to nests influences queen egg-laying during the pause. These queens were mated similarly as described above and were allowed to initiate egg laying and lay eggs until they entered the pause. Cessation of egg laying was visually confirmed for each queen, and they all entered the pause ~ 4–5 days later, similar to the timeframe observed in our primary experiment. Three days after entering the pause, brood was artificially added apart from the control group (see Fig. 1; Table 1). Of the initial 48 queens, 13 were removed from the dataset due to mortality before entering the pause, resulting in a final sample size of 35 queens for this experiment. These 35 queens were distributed into one of four treatment groups: (i) *control* (unmanipulated nests, no brood added; *n* = 9); (ii) *younger larvae added* (*n* = 5); (iii) *older larvae added* (*n* = 10); (iv) *late-stage brood added* (*n* = 11). For the brood addition treatment groups, clusters of worker brood, consisting of an estimated five individuals (range: 2–8), were carefully removed from a separate set of worker-producing colonies (*n* = 6) and placed in queen nests. After the manipulation, queens were monitored daily, noting the date of egg-laying reinitiation. Queens and their nests were collected after queens re-initiated egg laying. We dissected all nests at the end of the experiment to retroactively determine how much brood was added to each nest. Here, we used nest dissection methods similar to those described above for Experiment 2B.

### Statistical analyses

All statistical analyses were performed in R version 4.3.1 [47] and only p-values < 0.05 were considered significant. All data were non-normally distributed based on Shapiro-Wilk tests. For most of our analyses, we used generalized linear mixed models (GLMMs) to explore how different factors contributed to our experimental responses. Otherwise, chi-squared tests (to explore the influence of brood on queen egg laying) or Fisher’s Exact tests (to explore the influence of worker presence/absence on the number of eggs per cup and the total eggs at the end of the experiment) were used. We included the timing of certain nest development events (e.g., number of days before new egg cups are present), colony developmental stage, various treatments (e.g., worker presence/absence), brood type, and the duration of pause as possible fixed effects. Queen natal colony was always included in all models as a random effect. When biologically relevant (e.g., for analyses exploring ovarian resorption), body size and queen ID (nested random effect) were also included as random effects in models. GLMMs were performed with the glmmTMB functions in the R package glmmTMB [48]. We tested all possible models for each response variable based on all additive and interactive combinations of fixed effects while holding random effects constant. The best-fit model for our data was selected based on Akaike’s Information Criterion (AIC) using the “model.sel” command within the MUMIn package [49]. Following model selection, a one-way analysis of variance (ANOVA) was used to compare the data, and an Estimated Marginal Means (emmeans) post-hoc was performed to create pairwise comparisons. See Supplementary materials for more information regarding model selection (Table S1), best-fit model descriptions, and details regarding data selection.

For Experiment 1a, we calculated an average number of new egg cups per day across 45 consecutive days for all queens in the experiment (*n* = 62). To examine queen egg laying patterns across time, and to look for a period of cessation of egg laying, total new egg clutches were concatenated for individual queens in 5-day intervals (days 1–5, 6–10, 11–15, 16–20, and 21–25). We also related the duration of each queen’s pause to the following nest developmental characteristics: (i) the number of days until the first adult worker emerged in the nest (total range observed: 18–29 days); (ii) the total number of workers to emerge over a 7-day period, which likely represents the first cohort to emerge [50] (total range observed: 1–7 days); and (iii) the body sizes of all adult workers to emerge from the nest (total range observed: 1.45–3.0 mm; size was based on average of wing marginal cells as described above).

To analyze if worker presence is necessary for sustained and accelerated egg-laying (Experiment 2), we compared the accumulation of eggs for fifteen days post-worker emergence between the two treatments: (worker presence, worker+, *n* = 10) and worker absence (worker-, *n* = 20). For this analysis, queens were aligned such that day one was the first day workers were noted per individual nest, as the actual day of actual first worker emergence (range day 19–27 out of 45) varied per individual queen. The fifteen-day period post-worker emergence was determined based on this alignment and egg accumulation was determined in three five-day sequential segments across this period. According to the AIC score, the best-fit model included the additive interaction between the presence/absence of workers and the number of days after the first worker emergence. The second-best model (an interactive effect with an AIC score different by two) was chosen for this analysis because there is not likely to be a biologically relevant additive interaction between presence/absence of workers and the number of days after the first worker emergence.

For Experiment 3, we examined the terminal oocyte length (using only non-resorbed ovaries) and resorption status for all queens used in the experiment. There was no difference in terminal oocyte length and thus was not included in the remaining analyses (see Supplementary, Fig. 3).

For Experiment 4, all queens within the younger larvae group rejected the brood added to their nests, thus the five *younger larvae added* nests were excluded from the subsequent analyses. With the remaining queens, we used the number of days until egg-laying was re-initiated as our response variable. In our statistical analysis, we considered the amount of brood added to nests as a factor in our model selection, but the best-fit model excluded this factor, and thus only treatment was included in the analysis; see Supplementary Material.

For more information about the analyses completed for the manuscript, see Supplementary material, Table S2.

## Results

### Dynamic patterns of queen egg laying across the early nesting period

**Fig. 2:**
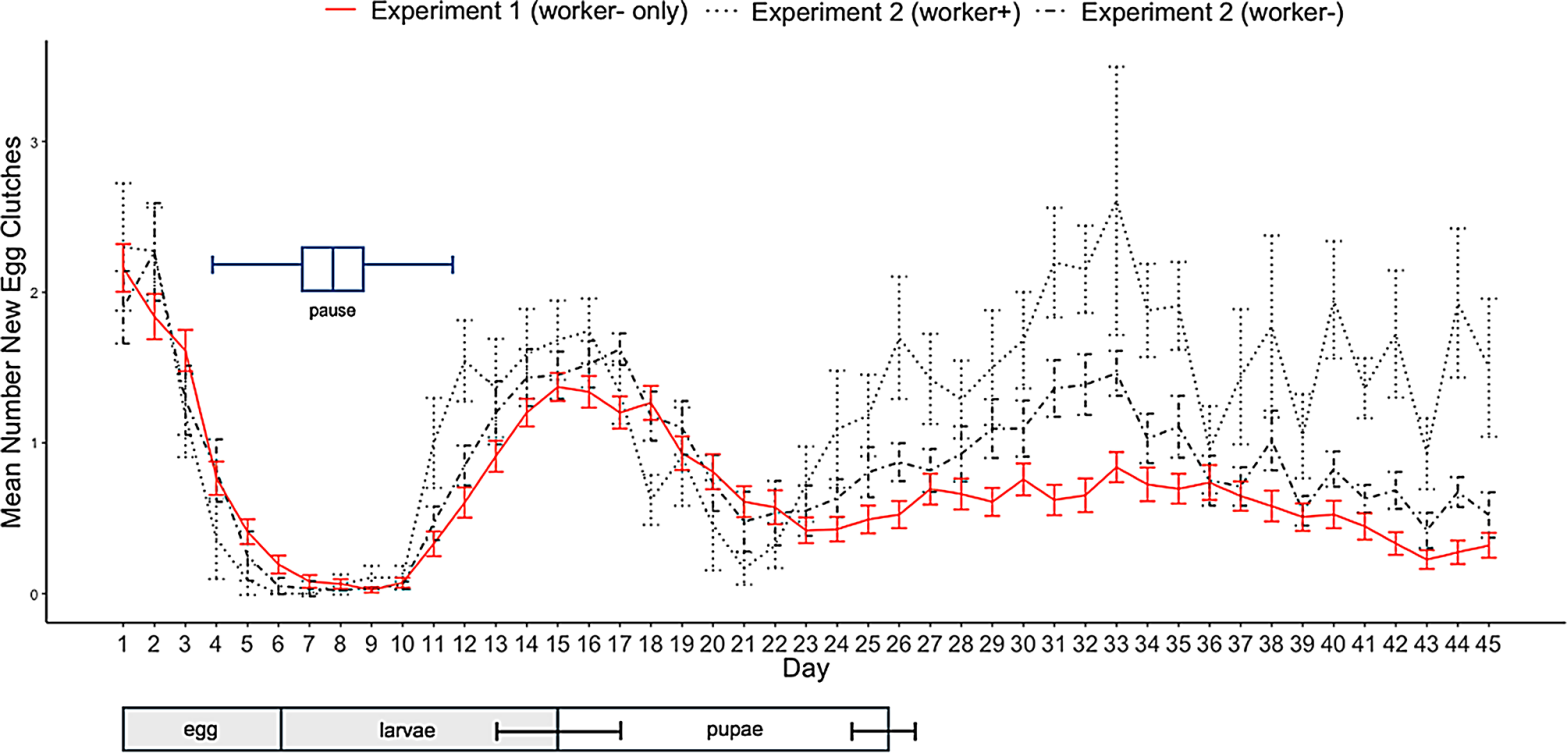
Differences in queen egg laying across five-day sequential periods in Experiment 1a. The X-axis represents five sequential, five-day periods in the experiment. The Y-axis shows the number of new egg cups observed in nests during each period. Box plots display median values (horizontal lines), interquartile ranges (boxes), and data variability (whiskers). Central dots represent means, and error bars indicate standard errors. Different letters above box plots denote statistically significant differences between periods based on an emmeans post-hoc pairwise test (*p* < 0.05)

**Fig. 3:**
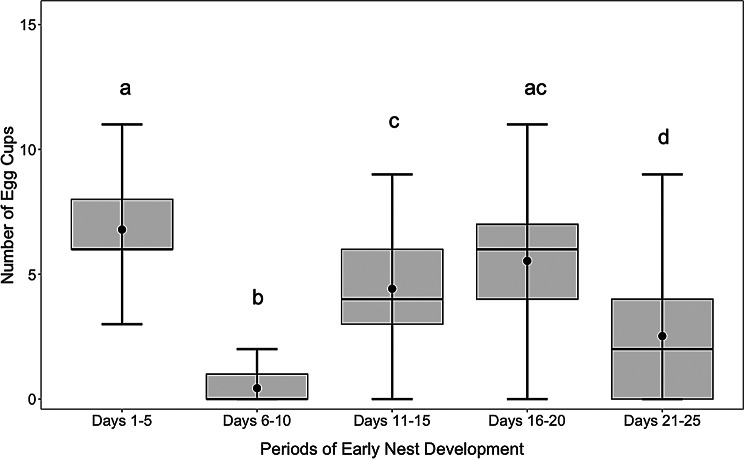
Patterns of queen egg laying across the early nesting stage (Experiments 1a and 2). Figure shows, across the 45-day experiment (X-axis), the daily mean (+/- s.e.m.) number of new egg cups present in nests (Y-axis) for all queens. Queens are separated into two lines according to the experiments: Experiment 1a data are shown in red, *n* = 62; Experiment 2 worker + is shown with the small, dotted line, *n* = 10, and Experiment 2 worker- is shown with the dot-dashed line, *n* = 20. Regardless of the experiment, all queens had a pause period, although the timing varied. A boxplot showing the duration of the pause for Experiment 1a is shown in blue, with the minimum = 4 days (left whisker), the median = 8 days, and the maximum = 12 days (right whisker). Below, the typical development times of *Bombus impatiens* workers (re-created and modified from Cnaani et al., 2002) are shown to demonstrate how the development of the first brood in the nest relates to our queen egg-laying data

All *B. impatiens* queens in Experiment 1a (*n* = 62) began laying eggs within an average of 28.18 ± 1.92 standard error of the mean (s.e.m) days. In all queens, the onset of egg laying was followed by a complete cessation in egg laying that began, on average, 4.21 ± 0.14 s.e.m. days after their first eggs were observed in the nest (Fig. 2). This cessation in egg laying lasted, on average, 7.74 ± 0.19 s.e.m. days, with a minimum length of four days and a maximum of 11 days, and always occurred between days 2–13 of the experiment. Hereafter, we refer to this cessation as a “pause”, defined as a complete cessation of egg-laying for two or more consecutive days. We define exiting the pause as then re-initiating egg laying for two or more consecutive days. When egg laying was compared across discrete five-day intervals before worker emergence, the lowest amount of egg laying occurred during the pause (which fell within the 6–10 day period; Fig. 3; GLMM χ² = 178.78, d.f. = 4, *p* < 0.001). During the interval that we refer to in this experiment as the pause period, as it best coincided with when most queens were in the pause, queens produced an average of 0.44 +/- 0.10 s.e.m. egg cups. This small number of eggs is likely due to the natural variation in the start and cessation of the pause, where the timing of the egg-laying period is variable across queens. There were also secondary declines in egg laying that occurred in the periods of days 11–15 (4.42 +/- 0.30 s.e.m. egg cups) and 21–25 (2.52 +/- 0.31 s.e.m. egg cups), but there was no complete cessation of egg laying observed in any queen other than during the pause period. During the first five-day interval (days 1–5), there was an average of 6.79 +/-0.26 s.e.m. new egg cups present, and during days 16–20, there was an average of 5.53 +/- 0.31 s.e.m. new egg cups. 100% of queens from the three other species that we observed (*B. vosnesenskii*, *B. mckayi*, *B. frigidus*) also exhibited the pause.

The pattern of queen reproductive development noted in Experiment 1a (encompassing both the pause and post-worker emergence) is further reflected in our analyses for Experiments 2 and 3. We found that the number of eggs in individual egg cups, as well as queen ovarian development and ovarian resorption across these timeframes, are dynamic according to social context. Pre-pause, queens produced an average of 1.48 +/- 0.07 s.e.m. (range of 1–4; *n* = 10 queens) eggs per egg cup. At the end of the experiment, worker- queens in Experiment 2 (*n* = 20) had an average of 5.15 +/- 0.26 s.e.m eggs in each cup, whereas queens with workers in the nest (*n* = 10) had an average of 5.66 +/- 0.22 s.e.m eggs in each cup; Fisher’s test to compare between treatments after 45 days, *p* = 0.4. At the end of the experiment (day 45), all queens with workers (worker+) had more egg cups (range: 1–9 cups, average = 5.6+/- 0.73 s.e.m.) than queens without any workers (worker-) (range: 1–7 cups, average = 2.45 +/- 0.49 s.e.m).

**Fig. 4:**
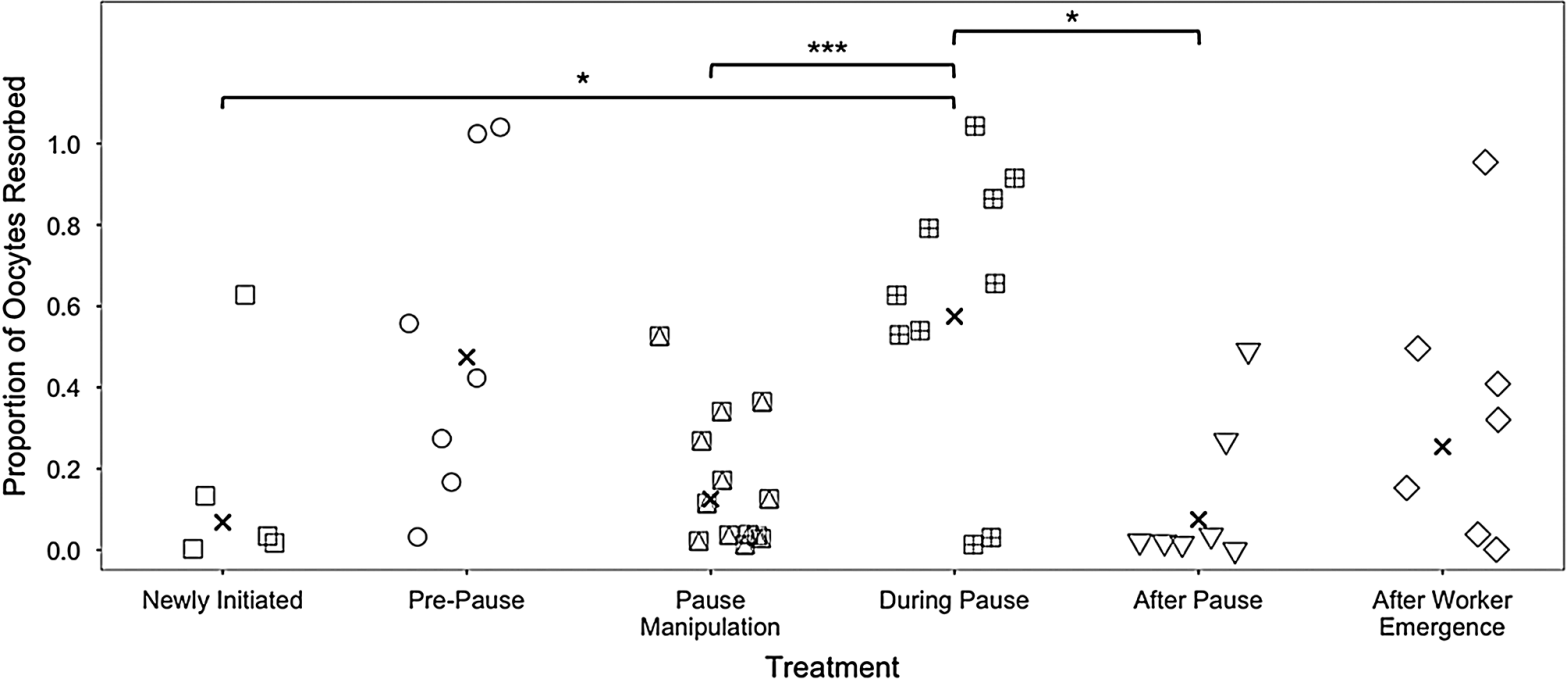
Resorption status of queen ovaries at different stages of colony development, or with brood artificially added to the nest (pause manipulation treatment) in Experiment 3 (*n* = 62). Shaped points represent the proportions of resorbed oocytes for individual queens, jittered to better visualize overlapping points. Each column represents a treatment with the X symbol amongst the jittered points representing averages for a given treatment group. An emmeans post-hoc pairwise test was used to determine differences between treatments. Brackets indicate statistically different means, with a black star (*) above them indicating a level of significance (* *p* < 0.05, ** *p* < 0.01, *** *p* < 0.001)

The highest ovarian resorption levels were observed immediately before and during the pause (Fig. 4; GLMM χ² = 32.54, d.f. =5, *p* < 0.0001; model included colony developmental stage). The greatest differences in the proportion of oocytes resorbed were in the *newly initiated* vs. *during pause* (Z = -3.21,*p* = 0.0169); *pause manipulation* vs. *during pause* (Z = -5.18, *p* < 0.0001), and *during pause* vs. *after pause* (Z = 3.11, *p* = 0.0231) (Fig. 4). No other groups differed significantly from each other (*pre-pause* vs. *pause manipulation*, Z = 2.81, *p* = 0.06).

### Influence of brood on queen egg laying

**Fig. 5:**
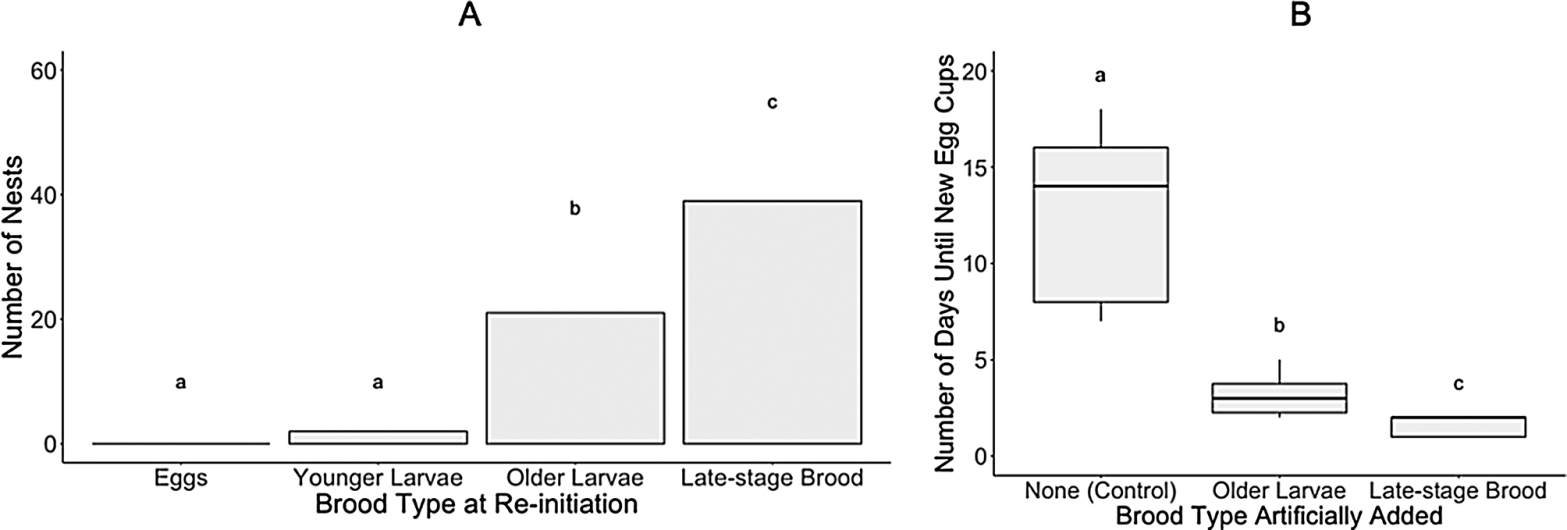
Influence of brood on queen egg-laying behavior. Part **A** (Experiment 1a): The oldest brood in the nest when queens exited the pause (i.e., re-initiated egg-laying), based on our first experiment (*n* = 62 queens). X-axis, the oldest brood type present in the nest at re-initiation; Y-axis, the number of nests. Part **B** (Experiment 4): Number of days until re-initiation of egg-laying in brood addition experiment (*n* = 30 queens). X-axis, brood type artificially added; Y-axis, the number of days between brood addition and the first day new eggs were observed in the nest. Means and standard error are shown. The letters represent significant differences between treatment groups based on emmeans pairwise comparison for both parts **A** and **B** of the figure

In Experiment 1a, queens always entered the pause after they had, on average 6.40 (+/- 0.19 s.e.m.) egg cups in their nests (*n* = 62), and were more likely to enter the pause when they only had eggs in the nest (71% of queens; *n* = 44) rather than young larvae in addition to eggs (29%; *n* = 18) (pairwise chi-square test, *p* < 0.001). Exiting the pause was also associated with social conditions in the nest. When queens exited the pause, they were more likely to have late-stage brood present as the oldest developed brood in the nest (63% of queens; *n* = 39), as opposed to having older larvae (34%; *n* = 21) or younger larvae (3%; *n* = 2), and no queens had only eggs when they exited the pause (Fig. 5A).

In our brood addition experiment (Experiment 4), queens took less time to re-initiate egg laying when we artificially added either *older larvae* (*n* = 10) or *late-stage brood* (*n* = 11) to the nests during the pause, relative to the *control* queens where no brood were added (*n* = 9) (GLMM χ² = 96.516, d.f. = 2, *p* < 0.001). Queens re-initiated within an average of 1.55 +/- 0.16 s.e.m days when late-stage brood were added, and within an average of 3.1 +/- 0.31 s.e.m days when older larvae were added, as compared to unmanipulated (control) queens, which took 12.5 +/- 1.46 s.e.m. days to reinitiate (Fig. 5B). Post-hoc pairwise comparisons using emmeans revealed significant differences among all groups: *control* versus *older larvae* (Z = 6.70, *p* < 0.001), *control* versus *late-stage* brood (Z = 7.96, *p* < 0.001), and *older larvae* versus *late-stage brood* (Z = 4.36, *p* < 0.001).) Regardless of what stage brood was added to nests, the number of days until re-initiation was four times faster for manipulated queens than queens in the control treatment. In all cases where older larvae (third instar and/or pre-defecation fourth instar) were added to the nests, these larvae had developed into late-stage brood (post-defecation fourth instar, prepupae, and/or pupae) by the time the queens re-initiated brood care.

### Association between the pause and nest developmental characteristics

All queens invariably paused in our study (see Fig. 3), so we examined whether the pause duration (which did vary; range of 4–11 days across all queens) was associated with any nest developmental characteristics using queens from Experiment 1a. There was a strong positive relationship between the length of the pause and the duration of time until the first worker emergence (GLMM χ²= 16.12, d.f. =1, *p* < 0.001). We did not find any association between the duration of the pause and either the number of workers to emerge within the first week (GLMM χ² = 0.9273, d.f. = 1, *p* = 0.3356) or the body size of all workers produced by nests (GLMM χ² = 0.0024, d.f. =1, *p* = 0.9609).

### Queen egg-laying is sustained by adult workers in the nest

**Fig. 6:**
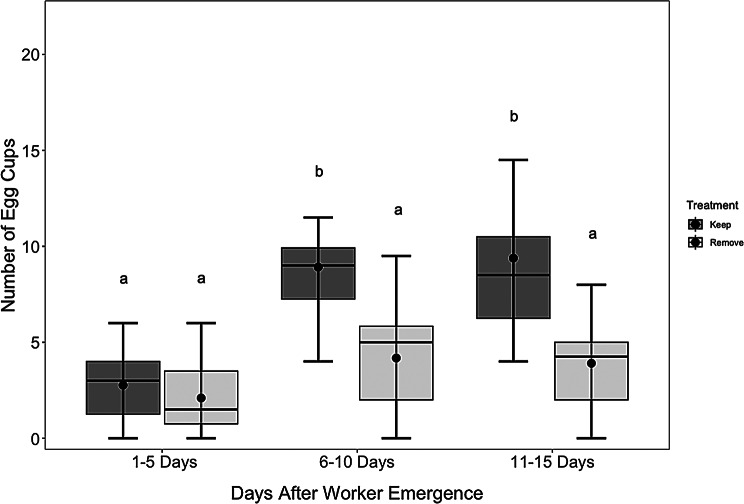
Differences in the number of egg cups between three distinct periods after worker emergence (Experiment 2). Data are presented for queens with workers present (worker+, *n* = 10) and nests with workers removed (worker-, *n* = 20). X-axis, time periods in five-day intervals; Y-axis, the number of new egg cups in the nest. Box plots display median values (horizontal lines), interquartile ranges (boxes), and data variability (whiskers). Central dots represent means, and error bars indicate standard errors. An emmeans post-hoc pairwise test was used to determine differences between the number of egg cups between treatment types (worker + vs. worker-) and between the five-day intervals. Different letters above box plots denote statistically significant differences between periods based on an emmeans post-hoc pairwise test (*p* < 0.05)

Lastly, in Experiment 2, we asked whether workers that emerge in young nests are required for queens to maintain high levels of egg laying by allowing workers to remain in some nests (*n* = 10; worker+) or removing them on the day each worker emerged (*n* = 20; worker-). On average, workers emerged 22.23 +/ 0.38 s.e.m. days after the first eggs were noted in the nest. Queens with workers present in the nest (worker+) had more egg cups compared with those that had no workers (workers-) at later intervals, with the greatest differences occurring 6–10 days (worker + average = 8.92 +/-0.96 s.e.m., worker- average = 4.19 +/-0.59 s.e.m.; emmeans pairwise comparison, Z = 3.80, *p* = 0.001) and 11–15 (worker + average = 9.38 +/-1.51 s.e.m., worker- average = 3.81 +/-0.44 s.e.m.; emmeans pairwise comparison, Z = 4.78, *p* < 0.001) days after the first worker emergence (Fig. 6). During the first five days (1–5 days) after worker emergence, the number of egg cups did not differ between the two groups (worker + average = 2.78+/-0.63 s.e.m., worker- average = 2.17 +/-0.39 s.e.m.; emmeans pairwise comparison, Z = 0.91, *p* = 0.361). Independently, the timing (χ²= 25.25, df = 2, p-value < 0.001) and treatment (χ²= 34.40, df = 1, p-value < 0.0001) were significant factors in the GLMM, but the interaction between the two factors was not.

The total number of brood (regardless of stage, including eggs, younger larvae, older larvae, and late-stage brood) at the end of Experiment 2 was also positively associated with the presence of workers. The average number of total brood, regardless of stage, in the nests of queens with workers was 126.9 +/- 12.92 s.e.m., relative to an average of 35.26 +/- 4.75 s.e.m. for nests with workers removed (GLMM: χ² = 470.55, d = 1 *p* < 0.001).

## Discussion

We demonstrated that in bumble bees, queen reproduction is flexible such that egg-laying accelerates and decelerates with changes in social conditions across nest development. Thus, social conditions prove to be a powerful influence on the pace of queen egg laying and overall nest growth. We demonstrated that both developing brood and adult workers socially control queen egg laying in *B. impatiens*, building on previous work [25, 26, 42, 51–53].

In contrast to the hyper-reproductivity observed in some eusocial systems, including fire ants (*Solenopsis invicta* [54, 55]), western honey bees (*Apis mellifera* [56]) and driver ants (*Dorylus wilverthi* [57]), solitary-founding queen egg laying occurs at a slower pace [8]. In solitary-founding systems, queens are non-reproductive for much of their adult life and only transition to egg-laying around the time that nest sites are located [8] (but see Sarro et al. [58]). In bumble bees, most of which follow such an annually social lifestyle, queens only reach their full reproductive potential (i.e., maximum rate of egg-laying) after the first adult workers emerge in the nest and assume brood care [25]. We found strong evidence that in *B. impatiens*, and suggestive evidence in additional bumble bee species (albeit with limited data), queens exhibit a stereotypical pattern where they reduce their egg-laying rate for several days soon after nest initiation, then accelerate it around the time they have late-instar larvae or pupae in the nest. This pattern, which we refer to as the “pause,” has been reported by previous bumble bee researchers [10, 12, 32] but has not been fully formally characterized or studied. The function and significance of the pause for queens and their nests are not currently known, but we propose that it allows queens to time their accelerated egg laying so that it coincides with the emergence of the first worker brood, who assume brood care duties in nests. If true, then the pause is a life history strategy that allows queens to match their reproductive timing to social conditions in the nest. This strategy might allow queens to minimize their performance of brood care and ultimately maximize egg-laying and nesting success.

We also found novel evidence that workers are required for queens to sustain higher rates of egg-laying beyond the earliest stages of nest initiation. Egg-laying rates in bumble bee queens never reach the same magnitude as egg-laying as in the aforementioned hyper-reproductive systems, but at the height of colony development queens can lay several cohorts of brood day and ultimately produce up to several hundred offspring in their nests [12, 59, 60]. In many animal systems, the number of subordinate individuals is positively associated with the number of offspring that the dominant or primary reproductive can produce (e.g., honey bees [61], *Myrmica* ants [62]; fire ants [54]; bumble bees [12]; meerkats [63]). With an increasing number of individuals available to perform care-related duties and foraging, the dominant or primarily reproductive individual is hypothesized to be able to specialize more fully in reproduction. Our work demonstrates that the switch to hyper-reproductivity in bumble bee queens is not permanent but instead is plastic, based on the presence of workers in the nest, or as seen previously, depending on the number of workers in the nest [42].

Two types of social group members positively influenced queen egg laying in our study: late-stage brood (older larvae and pupae) and adult workers. We note that younger brood may also positively impact queen egg laying, as we were not able to include them in our brood manipulation experiment; yet we found in our unmanipulated queens that only older brood were associated with resumed egg laying following the pause, suggesting that younger brood do not have an influence. Older brood and adult workers both reflect helpers in the nest, although they differ in whether they indirectly predict help in the nest (late-stage brood, which will eclose on the order of days and soon take on brood care tasks) or are a more direct signal of help (adult workers). Prior studies have shown that bumble bee queen egg-laying can be induced prematurely by adding a variety of group members to the nest. This includes adult workers [25, 26], pupae [28, 29–31], conspecific queens [64–68], workers of different bumble bee species [67, 68], and even honey bees [69]. The wide range of individuals and associated stimuli previously described suggests that queens are highly sensitive to a multitude of cues, some of which may be indicative of help or the potential of help in the nest.

Queens may be particularly sensitive to social cues from brood in the subsocial stage, as they interact closely with brood and bear the initial burden of carrying out care for brood in the nest. There are precedents for brood control over social dynamics in social insects, including queen bumble bees. In social Hymenoptera, brood pheromones regulate and elicit a broad range of behaviors related to reproduction and brood care [70]. In honey bees, larvae produce brood pheromone, which has a broad range of physiological and behavioral effects on workers. These effects include inducing worker foraging by reducing juvenile hormone production [71, 72] and reducing egg laying by inhibiting ovarian development [73]. In bumble bees, larvae can emit hunger signals that indicate their nutritional status and release brood-feeding behavior by workers in the nest [74, 75]. Brood in young bumble bee nests can also suppress circadian rhythmicity in nest-founding queens, resulting in queens performing brood care across a 24-hour cycle [76]. A recurring theme from these studies is that brood can alter traits in caregivers in context-dependent ways that support social cohesion and the growth of social colonies.

We also found evidence that queens reduce their egg-laying rate and begin to resorb their oocytes, right at the time that their first brood requires the most intensive and consistent need for care (i.e., when their first brood have reached the larval stage). The pause has been previously noted by Alford [32], who stated that after the first cohort of eggs are laid in bumble bee nests, additional egg cups are not typically observed until the first offspring have reached the pupal stage. Yet, neither Alford, nor other early bumble bee researchers who also noted this pattern [10, 12], articulated its significance for queens and their young nests. The pause seems to involve both a cessation of egg laying and ovarian resorption, and we did not see similar resorption rates to the same degree outside of the pause timeframe. The proximate mechanisms causing queens to temporarily halt (and even reverse) their ovarian development and egg-laying are unclear. Egg laying is limited by food availability and nutrition in many animal systems, including birds [77–79], fish [80–82], amphibians (83, 84), ants [85], and also in other bee systems [86], including solitary [87] and social (stingless bees) [88] species. Queens were well-fed in our experiments, so some non-nutritional cue is likely driving this stereotypical pattern. The driver or cue may be either internal to queens (for example, age-related changes in reproductive hormone production) or a social cue derived from very young brood (eggs or early instar larvae), as these are the only other social group members at this earliest stage in nest development. Ovarian resorption was highest during the pause in our study, which suggests that queens are reallocating resources away from ovarian development and toward other processes during this period [89–92]. Perhaps, as egg production can be costly, bumble bee queens may need to redirect resources to other processes during the pause, such as brood care [93]. Under natural conditions, the pause may also function to allow queens to direct more resources towards stressors, such as nutritional stress, parasites and/or pathogens, and pesticide exposure, rather than in egg production. These factors are known to play a crucial role in early nesting development [94–96].

All *B. impatiens* queens in our experiment performed the pause, with only the duration of the pause varying across the queens. The longer that *B. impatiens* queens paused in our study, the later their first workers emerged. The reason for this relationship is unclear, but it is generally consistent with a previous study in *B. impatiens* showing that queen brood care can, counterintuitively, delay offspring development times [50], although an opposite pattern has been observed in *B. terrestris* [42]. We also observed the pause in a limited data set for the closely related *Bombus vosnesenskii* (*n* = 6) and an even smaller set of additional species (*Bombus mckayi*, *n* = 2; *Bombus frigidus*, *n* = 1), and it has been mentioned more broadly in the literature and by bumble bee biologists (see Supplementary Material). We propose, therefore, that the pause does serve some purpose for young nests, which may be specifically related to how bumble bee queens balance brood care and egg laying during nest establishment.

## Conclusions

We provide further evidence that bumble bee nests, even at their most nascent developmental stage, contain multiple mechanisms of social regulation that coordinate the reproductive status and activities of social group members. We also present novel empirical data that queen egg laying does not follow a steadily increasing pattern, and instead, there are periods wherein queens reduce their egg laying that may relate to conditions within the colony. This is counter to the prevailing framework that social insect queens are continuously hyper-reproductive, which is true in some systems and at some colony developmental stages, but is not all-encompassing. Our work on the pause was inspired by early natural history descriptions of bumble bee nesting biology [10, 12, 32], demonstrating the value of detailed organismal observations as the foundation for more comprehensive studies. Our findings shed light on a life stage (solitary nest-founding) that is extremely under-studied in bumble bees and other eusocial insect systems. This early nesting stage may be especially important for population dynamics in solitary-founding species, as it is compulsory to the overall success of the colony. Social control of queen egg laying may allow queens to more fully realize both personal and nest fitness. We propose that complex mechanisms have evolved that support nesting success by placing queen reproduction at least partially under the control of social group members.

## Electronic supplementary material

Below is the link to the electronic supplementary material.

Supplementary Material 1

## Data Availability

All data and associated code are available through Dryad: http://datadryad.org/stash/share/PeHDPkT3LPRIjksr18ER2DVSFS0fBqOxItWRu1SULqk.
