# Supplementary material for: Social control of egg-laying in independently nest-founding bumble bee queens
Source: BMC Ecol Evol. 2025 Apr 9;25:30. doi: 10.1186/s12862-025-02364-0 (PMC11980249; doi:10.1186/s12862-025-02364-0)
Supplement: Supplementary file 1 — Supplementary Material 1 [file 12862_2025_2364_MOESM1_ESM.docx]

Table S1: Model Descriptions

| Model Description | Response | Factors | Estimate | Std. Error | z value | p-value | AIC |
| --- | --- | --- | --- | --- | --- | --- | --- |
| 5-day intervals and patterns of egg-laying  (e5kre) | Total # egg clutches | Timing:  (Days 6-10  Days 11-15  Days 16-20  Days 21-25)  + random factor “natal colony” | -2.72453  -0.37998  -0.117726  -0.90345 | 0.22103  0.08525  0.07841  0.13302 | -12.33  -4.46  -2.26  -6.79 | <2e-16  <2e-16  8.30e-6  0.0238  1.1e-11 | 1244.4 |
| Proportion of Ovaries resorbed (PropOVsub) | Proportion of ovaries resorbed | Colony developmental stage:  (pre-pause,  brood addition,  during pause,  after pause,  after worker emergence)  + random factor “natal colony”  + random factor “queen body size” | 1.2188  -0.6549  -2.1392  -0.0258  0.8881 | 0.7401  0.7408  0.6671  0.7967  0.6934 | 1.647  -0.884  3.207  -0.032  1.281 | 0.09959  0.37671  0.00134  0.97417  0.20027 | 78.2 |
| Brood Manipulation (Exit of Pause – MRR1s) | # days until re-initation of egg-laying (with brood added pre-maturely) | Brood type added:  (Older larvae  Late-stage brood)  +random factor  “natal colony” | -9.456  -11.010 | 1.412  1.383 | -6.698  -7.962 | 2.11e-11  1.70.1e-15 | 106.4 |
| Length of pause and first worker emergence (F1) | # days until first worker emergence | Length of pause  +random factor  “natal colony” | 0.485925 | 0.005596 | 86.83 | <2e-16 | 382.1 |
| Length of pause and # workers within one week | NA | NA | NA | NA | NA | NA | NA |
| Length of pause and worker body size | NA | NA | NA | NA | NA | NA | NA |
| # egg clutches Post-Worker emergence (MegKRE) | Total # egg clutches ~ | Treatment/Timing:  (Worker-,  6-10 days,  11-15 days  Worker-:6-10  Worker-: 11-15 )  +random factor  “natal colony” | -0.2684  1.0875  1.1266  -0.4096  -0.5740 | 0.2936  0.2685  0.2664  0.3438  0.3403 | -0.914  4.051  4.229  -1.191  -1687 | 0.3607  5.10e-05  2.35e-05  0.2336  0.0916 | 420.9 |
| Total Brood (Totbrood1) | Total # brood ~ | Treatment:  (worker-)  +random factor  “natal colony” | -85.953 | 3.962 | -21.69 | <2e-16 | 483.8 |

Best-fitting models:

| Patterns of egg-laying across 5-day intervals | | | |
| --- | --- | --- | --- |
| **Model** | **TM** | **df** | **AIC** |
| Null | NA | 4 | 1244.4 |
| **eKRE1** | **+** | **8** | **1465.1** |

Included TM, i.e., timing of nest development and source colony as a random factor. The best model fitting our data was selected based on the Akaike’s Information Criterion (AIC). (+) parameters included in the model, and NA, not included parameters. The selected best model is given in bold.

| Resorption of ovaries | | | |
| --- | --- | --- | --- |
| **Model** | **T** | **df** | **AIC** |
| Null | NA | 6 | 95.8 |
| **OVQ1** | **+** | **11** | **78.2** |

Included T, i.e., treatment of queen nests (when artificial brood addition occurred) as fixed factors and source and queen body size as random factors. The best model fitting our data was selected based on the Akaike’s Information Criterion (AIC). (+) parameters included in the model, and NA, not included parameters. The selected best model is given in bold.

| Length of Non-Resorbed Terminal Oocytes | | | |
| --- | --- | --- | --- |
| **Model** | **T** | **df** | **AIC** |
| **Null** | **NA** | **4** | **1037.5** |
| OVresorbN1 | + | 9 | 1041.7 |

Included T, i.e., treatment of queen nests (when artificial brood addition occurred) and source and queen body size as random factors. The best model fitting our data was selected based on the Akaike’s Information Criterion (AIC). (+) parameters included in the model, and NA, not included parameters. The selected best model is given in bold.

| Artificial Brood Manipulation | | | | | |
| --- | --- | --- | --- | --- | --- |
| **Model** | **T** | **B** | **T:B** | **df** | **AIC** |
| Null | NA | NA | NA | 3 | 166.3 |
| **MRR1s** | **+** | **NA** | **NA** | **5** | **106.4** |
| MRR2s | NA | NA | + | 5 | 124.5 |
| MRR3s | NA | NA | * | 6 | 127.5 |
| MRR4s | NA | + | NA | 4 | 147.2 |

Included T i.e. Treatment type (type of brood added), B i.e. Number of brood added as fixed factors and source colony as a random factor. The best model fitting our data was selected based on the Akaike’s Information Criterion (AIC). (+) parameters included in the model, and NA, not included parameters. The selected best model is given in bold.

| Length of pause and first worker emergence | | | |
| --- | --- | --- | --- |
| **Model** | **L** | **df** | **AIC** |
| Null | NA | 4 | 224.4 |
| **F1** | **+** | **5** | **235.6** |

Included L i.e. Length of the pause and source colony for queens as a random factor. The best model fitting our data was selected based on the Akaike’s Information Criterion (AIC). (+) parameters included in the model, and NA, not included parameters. The selected best model is given in bold.

| Total # of workers within first week | | | |
| --- | --- | --- | --- |
| **Model** | **L** | **df** | **AIC** |
| **Null** | **NA** | 4 | 204.6 |
| KWQ1 | + | 5 | 207.0 |

Included L i.e length of the pause and source colony for queens as a random factor. The best model fitting our data was selected based on the Akaike’s Information Criterion (AIC). (+) parameters included in the model, and NA, not included parameters. The selected best model is given in bold.

| Worker body size and length of pause | | | |
| --- | --- | --- | --- |
| **Model** | **L** | **df** | **AIC** |
| **Null** | **NA** | **4** | **24.8** |
| WB1 | + | 5 | 26.9 |

Included L i.e length of the pause and source colony for queens as a random factor. The best model fitting our data was selected based on the Akaike’s Information Criterion (AIC). (+) parameters included in the model, and NA, not included parameters. The selected best model is given in bold.

| Number of egg clutches post-worker emergence | | | | | |
| --- | --- | --- | --- | --- | --- |
| **Model** | **T** | **D** | **T:D** | **df** | **AIC** |
| Null | NA | NA | NA | 4 | 464.5 |
| MegKRETime1 | + | NA | NA | 5 | 445.3 |
| MegKRETime2 | NA | + | NA | 6 | 446.5 |
| **MegKRETime3** | **NA** | **NA** | **+** | **7** | **421.1** |
| MegKRETime4 | NA | NA | * | 9 | 423.1 |

Included T i.e. treatment (worker-/worker+) in nest, D, timing of nest development (# days after the first worker emergence) and source colony for queens as a random factor. The best model fitting our data was selected based on the Akaike’s Information Criterion (AIC). (+) parameters included in the model, and NA, not included parameters. The selected best model is given in bold.

Note: As an additive interaction may not be biologically relevant, the next best fit model (MegKRETime4) was used for the analysis.

| Total # brood and worker presence/absence | | | |
| --- | --- | --- | --- |
| **Model** | **T** | **df** | **AIC** |
| Null | NA | 2 | 1069.5 |
| **TotBrood1** | **+** | **3** | **484.7** |

Included T i.e. treatment (worker-/worker+) in nest and source colony for queens as a random factor. The best model fitting our data was selected based on the Akaike’s Information Criterion (AIC). (+) parameters included in the model, and NA, not included parameters. The selected best model is given in bold.

**Pause in other species:**

We observed three other species (*B. vosnesenskii*, *B. mckayi*, *B. frigidus*) to determine if the pause occurred. See methods of Experiment 1b for more details.

For *Bombus vosnesenskii*, we collected 12 queens in total. Out of the 12, 8 queens initiated normally, but 2 were removed due to lack of consistent photo data collection. Of the 8 total queens, 6 initiated normally, and thus data was collected. For *B. mckayi* and *frigidus*, we had limited specimens, and only three nests were available for observation.

Additionally, we spoke with Omar Arguello from El Colegio de la Frontera Sur, Chiapas, Mexico, who confirmed that this pattern occurred in the tropical bumble bee species *Bombus ephippium* (personal communication).


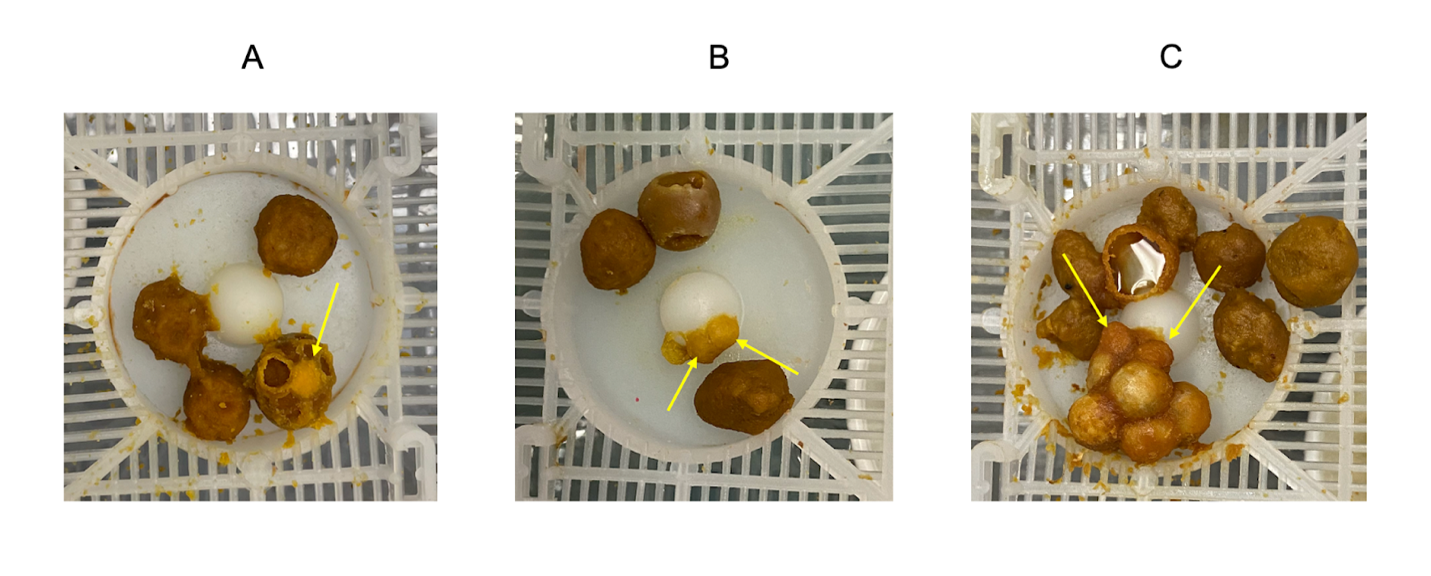


Supplementary Figure 1: **Example images of egg cups.** Photos show examples of when the first egg cups in the nest are laid directly on a pollen ball (A), directly onto the central plastic mound that is part of the nest box (B), or after pause, re-initiation on brood present (C). Individual egg cups are denoted with an arrow in photos. Other items in nests include pollen balls provided to nests (seen in all photos) and honey pots (in C only).


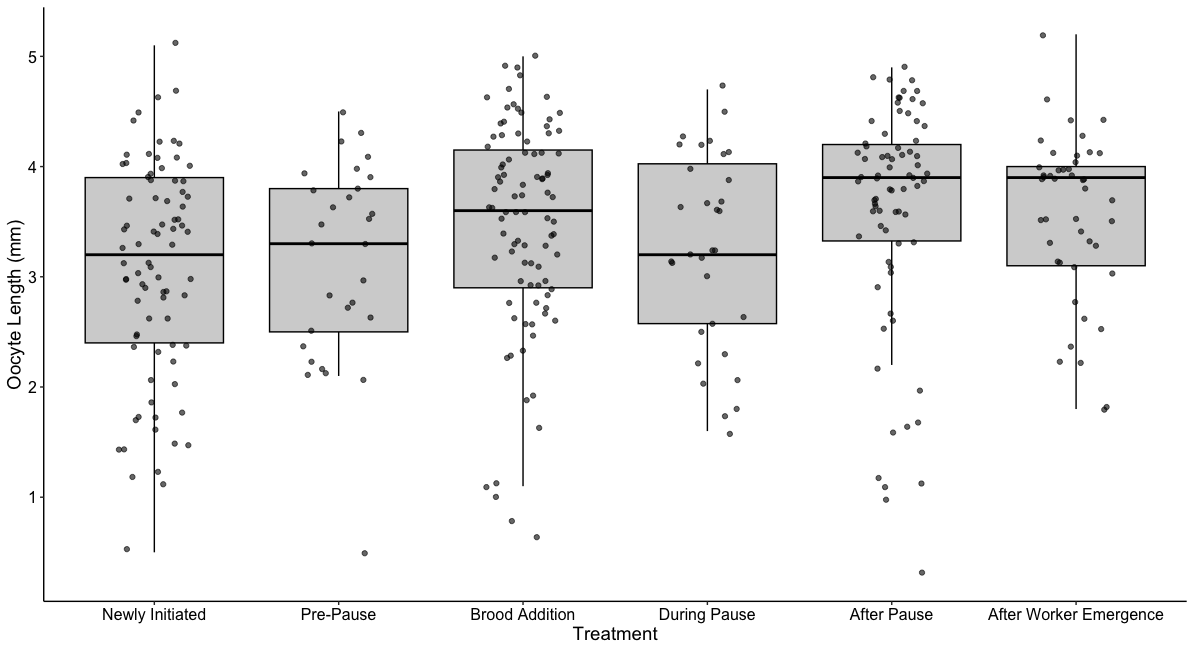


Supplementary Figure 2: **Terminal Oocyte Length.** Length of terminal oocytes measured per treatment type. Of the 62 queens used in the experiment, those without all 8 terminal oocytes present were excluded from the analysis. Thus, a subset of queens used in the experiment (n = 56) were analyzed. Treatment (when artificial brood addition occurred) was not a significant factor in the GLMM model selection and thus was not included.

Table S2: Analyses for Manuscript

| **Phenomenon** | **Question** | **Exp.** | **N=** | **Result** | **Type of Analyses** | **Family/**  **Distribution** | **Fixed Effects** | **Response Variable** | **Random Factors** |
| --- | --- | --- | --- | --- | --- | --- | --- | --- | --- |
| Dynamic patterns of queen egg-laying across early nesting period | 5-day intervals and patterns of egg-laying  (e5kre) | 1a | 62 | Sig | GLMM | Zero-inflated negative binomial | Timing | # of egg clutches | Natal colony |
| Dynamic patterns of queen egg-laying across early nesting period | Proportion of ovaries resorbed in relation to treatment | 3 | 62 | Sig | GLMM | ordbeta | Treatment | Proportion of ovaries resorbed | Natal colony & body size |
| Influence of brood on queen egg-laying | Brood addition and timing of re-initiation | 4 | 30 | Sig | GLMM | Gamma | Treatment | # days until re-initiation | Natal Colony |
| Influence of brood on queen egg-laying | Differences between brood type in nests (pause entry) | 1a | 62 | Sig | Pairwise chi-squared test | n/a | n/a | Oldest brood type present in the nest | n/a |
| Influence of brood on queen egg-laying | Differences between brood type in nests (pause exit) | 1a | 62 | Sig | Pairwise chi-squared test | n/a | n/a | Oldest brood type in the nest | n/a |
| Association between the pause and nest developmental characteristics | Length of the pause and # days until first worker emergence | 1a | 62 | Sig | GLMM | Gaussian | Length of Pause | # days until first brood emergence | Natal colony |
| Association between the pause and nest developmental characteristics | Length of the pause and # of workers within one week | 1a | 62 | Null | GLMM | n/a | n/a | # of workers within one week | n/a |
| Association between the pause and nest developmental characteristics | Length of the pause and the body size of all workers after emergence | 1a | 62 | Null | GLMM | n/a | n/a | Body size of workers | n/a |
| Queen egg-laying is also under the control of adult workers in the nest | Differences in the number of egg clutches after worker emergence | 2 | 30 | Model overall not sig. | GLMM | Zero-inflated negative binomial | Treatment* Timing | Total # egg clutches | Natal colony |
| Queen egg-laying is also under the control of adult workers in the nest | # of brood in nests and presence/  absence of workers in nest | 2 | 30 | Sig | GLMM | Poisson | Treatment | Total # brood | Natal colony |

Table 2: Summary of the analyses of related experiments examining queen egg-laying dynamics and nest development. N refers to the number of queens or nests involved in the experiments. n/a refers to not being included either because of the analysis in question (chi-square tests do not include the same information as GLMMs) or because the results were not significant in the model and thus not included.
